# Effect of Drought on Agronomic Traits of Rice and Wheat: A Meta-Analysis

**DOI:** 10.3390/ijerph15050839

**Published:** 2018-04-24

**Authors:** Jinmeng Zhang, Shiqiao Zhang, Min Cheng, Hong Jiang, Xiuying Zhang, Changhui Peng, Xuehe Lu, Minxia Zhang, Jiaxin Jin

**Affiliations:** 1 International Institutes for Earth System Science, Nanjing University, Nanjing 210023, China; beike0922@163.com (J.Z.); zhangshiqiao3039@163.com (S.Z.); chengmin007008@163.com (M.C.); lzhxy77@163.com (X.Z.); luxh@nju.edu.cn (X.L.); 18357173715@163.com (M.Z.); 2 Jiangsu Provincial Key Laboratory of Geographic Information Science and Technology, Nanjing University, Nanjing 200023, China; 3 School of Forestry & Bio-Technology, Zhejiang A&F University, Lin’an 311300, China; 4 Institute of Environment Sciences, Department of Biology Sciences, University of Quebec at Montreal, Montreal, QC H3C 3P8, Canada; peng.changhui@uqam.ca; 5 School of Earth Sciences and Engineering, Hohai University, No. 8 Fo cheng xi Road, Nanjing 211100, China; jiaxinking@126.com

**Keywords:** drought, crop, yield, agronomic traits, meta-analysis

## Abstract

Drought has been one of the most important limiting factors for crop production, which deleteriously affects food security worldwide. The main objective of the present study was to quantitatively assess the effect of drought on the agronomic traits (e.g., plant height, biomass, yield, and yield components) of rice and wheat in combination with several moderators (e.g., drought stress intensity, rooting environment, and growth stage) using a meta-analysis study. The database was created from 55 published studies on rice and 60 published studies on wheat. The results demonstrated that drought decreased the agronomic traits differently between rice and wheat among varying growth stages. Wheat and rice yields decreased by 27.5% and 25.4%, respectively. Wheat grown in pots showed greater decreases in agronomic traits than those grown in the field. Rice showed opposite growing patterns when compared to wheat in rooting environments. The effect of drought on rice increased with plant growth and drought had larger detrimental influences during the reproductive phase (e.g., blooming stage, filling stage, and maturity). However, an exception was found in wheat, which had similar decreased performance during the complete growth cycle. Based on these results, future droughts could produce lower yields of rice and wheat when compared to the current drought.

## 1. Introduction

Drastic climate changes and increased water scarcity challenge global food security, which is further exacerbated due to the need to feed a growing global population [1]. A reviewed estimate states that global agricultural production might need to increase by 60–110% to meet the increasing demands [2] as well as to provide food security to the predicted 870 million people who will be chronically undernourished by 2050 [3]. However, the rates of global crop production are far below the amounts required to meet projected demands by 2050 [4,5]. Crop yield is affected by agronomic factors and various environmental variables such as water availability and temperature [6,7]. There is extensive crop yield variability in many semi-arid regions, which are owed to water limitation and year-to-year fluctuations in meteorological conditions. Although an increase in temperature is beneficial for crop productivity in some cooler regions of the world, drought still significantly reduces national cereal production by 9–10% on a global scale [1] via negative effects on plant growth, physiology, and grain development [8,9,10]. Caused by reduced precipitation and increased temperature [11], drought has been the most important limiting factor for crop productivity and, ultimately, for food security worldwide [12]. Wheat (*Triticum aestivum* L.) and rice (*Oryza sativa* L.) are two of the three major global cereals (wheat, rice, and maize (*Zea mays* L.)) with global annual productions of 713.2 and 745.7 million tons, respectively. Wheat and rice contributed more than 50% to global cereal production in 2013 [13]. Previous studies have revealed that their grain yields have declined in recent years because of drought. These studies have included several meta-analyses, summary studies, and model simulation results with only drought taken into account or an interaction with temperatures (see Table 1). It will be necessary to increase biomass production and economic yield under the conditions constrained by climate and water availability [14]. However, before this can occur, the extent of wheat and rice yield reduction and other agronomic traits that are affected by changes in the climate must be understood.

Now, more than ever, the effect of drought on agronomic traits of rice and wheat has become more significant. With a changing climate, droughts are predicted to become more intense and frequent in many regions [19] with global agricultural drought frequency and area increasing by 50% to 200% during the 21st century so far [20]. Therefore, a number of independent studies have investigated the individual effects of drought on wheat and rice agronomic traits during different growth stages. The severity and duration of drought stress determine the extent of the yield loss by shortening the life cycle and duration of grain filling [8]. Leilah et al. [21] found that the panicle number, grain weight, and biological yield were the most effective variables that influenced grain yield in a wheat cultivar. Grain yield and yield components of rice have also been examined in relation to drought occurring at various stages of growth [22]. A change in any agronomic traits under drought conditions alters the final crop yield including the growth parameters (e.g., plant height and biomass at harvest) and components of harvestable yield such as the panicle number per unit area (PNPU), the grain number per panicle (GNPP), 1000-grain weight (GW), the panicle length (PL), and the filled grain percentage/seed setting rate (FP). However, the effect of these parameters across different crop groups and drought conditions has not been fully elucidated. Nonetheless, this information is required for developing agricultural practices aimed at minimizing the effect of drought. Furthermore, this information would improve the accuracy of crop-simulation models to evaluate the effects of projected climate change on crop yields.

The diversity of drought effects on agronomic traits of rice and wheat are too complex to compare using traditional methods. Therefore, meta-analysis was used in the present study to identify general trends among numerous independent drought-related studies and combine the results into one measure known as the effect size [23]. Meta-analysis has been used to study the response of wheat and rice to elevated O_3_ and CO_2_ [24,25] levels as well as to synthesize a crop production response to drought on a global scale [12,13]. In the present study, we conducted a meta-analysis assessment to summarize and synthesize the results of the numerous studies for agronomic traits such as growth as well as grain yield and its components of rice and wheat in response to drought. Furthermore, we explored the sources of variation among these two crops in response to drought. Our aim was to answer the following questions. To what extent is rice and wheat grain yield reduced by drought and how do the yield components respond to drought? Are the responses to drought dependent on growth stages, the rooting environment, and drought stress intensities? Do the wheat types lead to differences in wheat agronomic traits? Hopefully, the results can improve our quantitative understanding of the effect of drought on rice and wheat and help minimize the influence of drought on crop production. This will improve the accuracy of crop-simulation models that are used to evaluate the effects of projected climate change on crop yields.

## 2. Materials and Methods

### 2.1. Data Collection

Using the Web of Science (Thompson-ISI, Philadelphia, PA, USA) and the China Knowledge Resource Integrated Databases, a survey on the wheat and rice response to drought from 1980 to 2017 was completed. The database was built based on keywords such as wheat or rice, water stress, water deficit, drought, growth, yield, and productivity. Only studies that met the following selected criteria were included in the database: (1) involved plants that experienced drought under field conditions and pot studies, (2) the reported effect data of a water deficit was taken from the experiments including two datasets of a well-watered control group and a drought condition treatment group without other treatments (e.g., addition of fertilizer, modification of temperature, CO_2_ or O_3_), (3) contained any of the plant growth parameters of rice and wheat listed in Table 2, and (4) included replication in the experimental design. Data from the figures were digitized using data extraction software (Plot Digitizer-Windows). In total, 120 cultivars from 60 published wheat studies and more than 70 cultivars from 55 published rice studies were used in the global meta-analysis, which excluded all additional treatments (see Table S1).

The magnitude of crop responses to drought was examined based on the following categorical variables: (i) cereal species (wheat and rice), (ii) agronomic traits (see Table 2), (iii) drought timing or growth stages (see Table 2; productive phase included blooming and grain filling stages while the vegetative phase included jointing and booting stages); (iv) wheat types (winter wheat versus spring wheat); (v) drought stress intensity (mild, moderate, and severe), relative soil water content (RSWC), or soil water potential (SWP); and (vi) root environment (plants grown in the field, plot, and pot). These groups were used as moderators in the meta-analysis to distinguish effect size values.

For the meta-analysis in the present study, we focused on the effect of drought on rice and wheat agronomic traits. Therefore, only selected studies with the single effect of water deficit were included. We only selected data from the studies with paired groups (i.e., control and treatment) and, therefore, we considered that other environmental factors (e.g., temperature, light intensity, and CO_2_ concentration) were the same between the control and drought conditions. Drought intensity was classified into three levels (mild, moderate, and severe) based on the description in the studies. If drought intensity had not been elaborated on in the study, it was classified based on the RSWC including mild stress (55% < RSWC < 70%), moderate stress (35% < RSWC < 55%), and severe stress (RSWC < 35%) and according to the SWP (mild stress (−35 kPa < SWP < −10 kPa), moderate stress (−55 kPa < SWP < −35 kPa), and severe stress (SWP < −55 kPa)).

### 2.2. Meta-Analysis

The effect size for each observation was calculated as the natural log of the response ratio (R) to represent the magnitude of the responses of agronomic traits to drought [23,26].

(1) $$R=\ln\left(X_{e}/X_{c}\right)=\ln\left(X_{e}\right)-\ln\left(X_{c}\right)$$

where X_e_ and X_c_ are the mean response values of each individual observation in the treatment and control groups, respectively. Not all of the studies reported the mean values for the treatment and control conditions by reporting standard deviations or standard error values. Therefore, we used an unweighted meta-analysis as described in previous studies to maximize the number of observations in the studies [27]. The mean effect size of each categorical subdivision was calculated by the equation below.

(2) $$\bar{R}=\sum \left(R\right)/N$$

where N is the number of samples. Then the $\bar{R}$ was reported as the percent change (D, %) [25], which is more visible than $\bar{R}$ and was calculated by the equation below.

(3) $$D\left(\%\right)=\left(e^{\bar{R}}-1\right)\times 100\%$$

If the 95% confidence intervals (CI) of the percent change do not overlap with zero [24,25], this represents that the drought had a significant effect on the agronomic traits. The heterogeneity (Q_b_) for each categorical variable was determined for the response variable (see Table 3). The significant Q_b_ value indicated that the effect size was different between different agronomic traits. Figures were graphed using the Origin Pro 9.0 software program in Microsoft Windows.

## 3. Results

### 3.1. Overall Effects of Drought on Wheat and Rice

**Figure 1:**
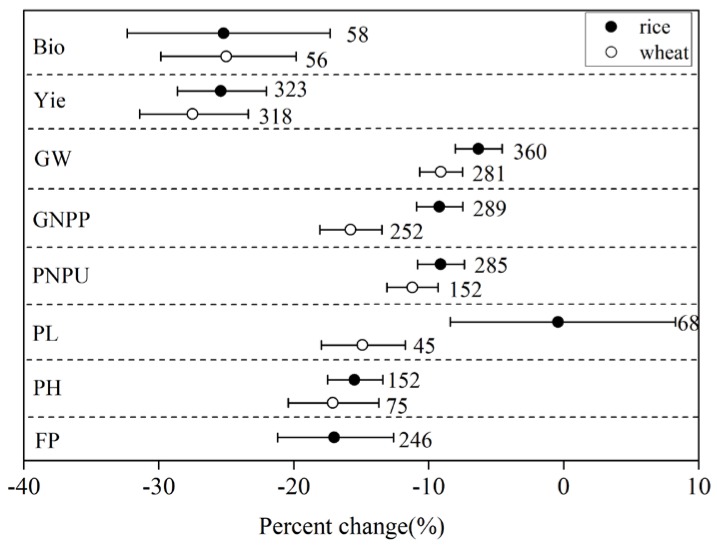
Effect of drought on the agronomic traits of rice and wheat. Numbers near the symbols specify the number of data points and the error bars indicate a 95% confidence interval. Abbreviations for the agronomic traits are described in Table 2.

Across all the studies, drought significantly decreased the agronomic traits of wheat and rice (see Figure 1) with biomass and yield showing the largest decreases. Drought decreased wheat biomass and yield by 25.0% and 27.5%, respectively, and decreased rice biomass and yield by 25.2% and 25.4%, respectively. The large yield loss in wheat was caused by a combination of decreases in GW (−10.6%), GNPP (−15.8%), PNPU (−11.2%), and PL (−14.9%). Similarly, in rice, the large yield loss was caused by a combination of decreases in GW (−6.3%), GNPP (−9.2%), PNPU (−9.1%), PL (−0.4%), and FP (−17.0%). Significant differences were found only in PL and GNPP between wheat and rice (see Figure 1). However, the drought decreased wheat growth more than rice growth in most agronomic traits except for the FP that had no comparison data.

### 3.2. Effect of Stress Intensity on Rice and Wheat in Response to Drought

**Figure 2:**
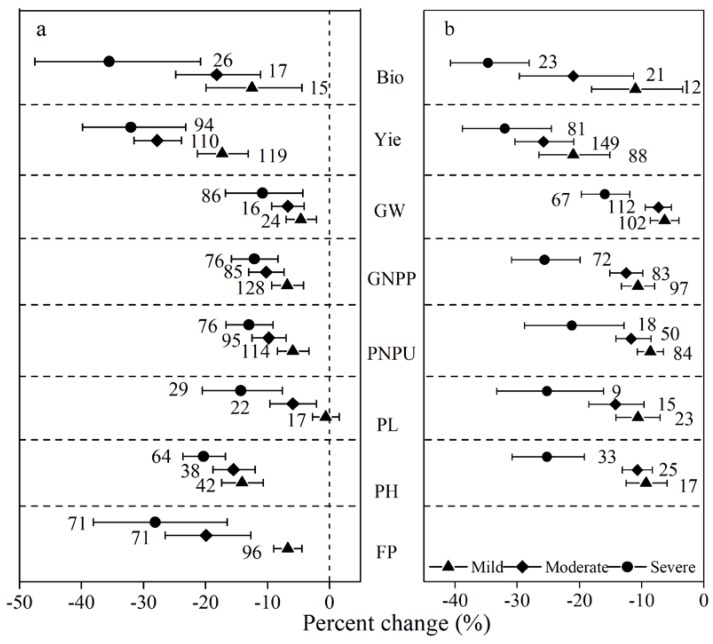
Response of rice and wheat to drought under different stress intensities. Numbers near the symbols specify the number of data points and the error bars indicate a 95% confidence interval. Abbreviations for the agronomic traits are described in Table 2 (**a**) Rice, (**b**) Wheat.

The detrimental effect of drought on agronomic traits was progressively greater as stress intensity increased with no exceptions in both rice and wheat (see Figure 2). The reductions of rice biomass under mild, moderate, and severe stress were 12.5%, 18.0%, and 35.5%, respectively (see Figure 2a). Additionally, the reductions in wheat biomass were 11.0%, 21.0%, and 34.7%, respectively (see Figure 2b). Drought decreased rice yield under mild, moderate, and severe stress by 17.0%, 27.8%, and 32.0%, respectively. Drought also decreased wheat yield by 21.0%, 25.8%, and 32.0%, respectively. Other variables such as FP, PL, GNPP, PNPU, and GW showed significantly larger reductions under severe stress than under mild stress. However, the GNPP, GW, and PH of wheat showed no differences between mild stress and moderate stress.

### 3.3. Effect of the Rooting Environment on Rice and Wheat in Response to Drought

**Figure 3:**
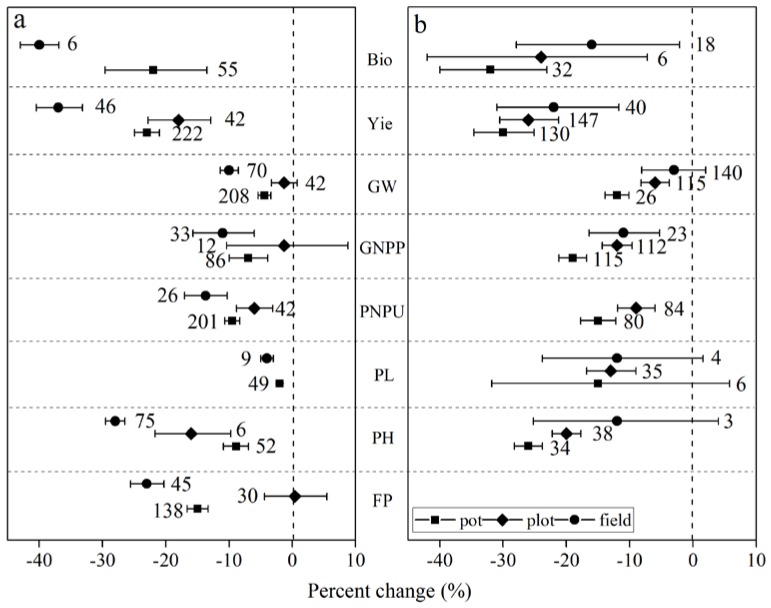
Response of rice and wheat to drought in different rooting environments. Numbers near the symbols specify the number of data points and the error bars indicate a 95% confidence interval (CI). Abbreviations for the agronomic traits are described in Table 2 (**a**) Rice, (**b**) Wheat.

From Figure 3, rice plants grown in the field showed the largest reductions in agronomic traits than rice plants grown in pots or plots in the greenhouse. Rice biomass and yield decreased the most by 40.0% and 37.4%, respectively, when grown in the field. However, rice showed the smallest reductions in yield, GW, GNPP, and PNPU (−6.0%, −3.0%, −1.3%, and −6.0%, respectively) when they were grown in plots (see Figure 3a). In contrast, wheat agronomic traits (e.g., biomass, yield, GW, GNPP, and PL) decreased the most (−32.5%, −30.3%, −12.0%, −19.5%, and −15.6%, respectively) and the least (−16.6%, −22.6%, −3.4%, −11.4%, and −12.0%, respectively) when they were grown in pots and in the field, respectively (see Figure 3b). However, we could not rule out the systematic errors of PH and PL because there were too few sample sizes (see Figure 3b).

### 3.4. Effect of Growth Stages on Rice and Wheat in Response to Drought

**Figure 4:**
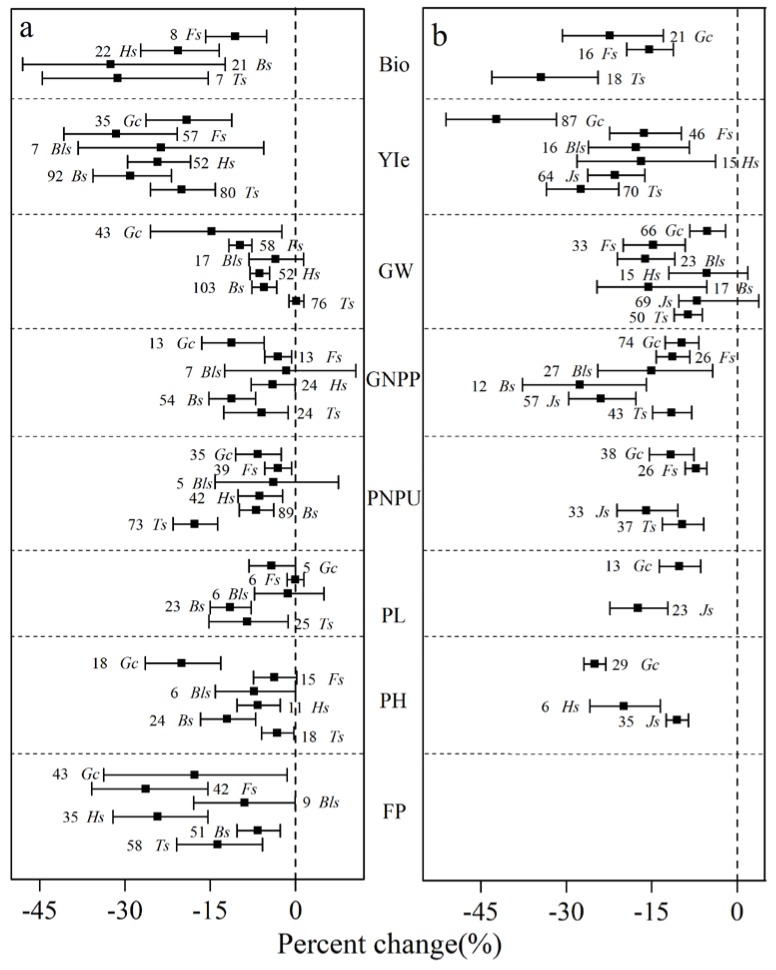
Response of rice and wheat to drought at different growth stages. Numbers near the symbols specify the number of data points and the error bars indicate a 95% confidence interval.

The effect of growth stages on rice varied among the variables (see Figure 4a). Rice biomass showed larger reductions during the tillering stage (−31.0%) and booting stage (−32.5%) under drought conditions. The smallest reductions of yield and GW occurred during the tillering stage (−20.0% and −0.2%, respectively) and the largest occurred during the filling stage (−31.5% and −9.7%, respectively) in addition to the decrease in GW (−14.7%) during the complete growth cycle. There were diminishing decreases in rice GNPP and PNPU with rice growth, which shows the largest decreases during the tillering and booting stages but the smallest decreases during the filling stage. Droughts that occurred during the booting stage had the greatest negative effect on rice PL. Rice PH decreased the most (20.0%) during the complete growth cycle and decreased by 3.2% during the tillering stage. FP decreased the most (26.3%) during the filling stage.

Most variables of wheat (see Figure 4b) showed significant decreases during different growth stages. Only GW did not decrease significantly during the jointing stage and heading stage under the drought condition with 95% confidence intervals overlapping with zero. Drought decreased wheat biomass the most by 34.4% at the tillering stage. Drought decreased wheat yield the most during the complete growth cycle, which was achieved from a combination of decreases during the tillering stage (−27.4%), the jointing stage (−21.4%), the heading stage (−16.8%), the blooming stage (−17.7%), and the filling stage (−16.3%). Biomass, PNPU, GUPP, and GW did not indicate a clear changing trend with the growth and development of wheat. Wheat PNPU showed the largest decrease (−15.8%) during the jointing stage and the smallest decrease (−7.0%) during the filling stage, which owes to drought. Wheat GNPP showed the largest decrease (−27.6%) during the booting stage. Wheat PH decreased the most (−25.0%) during the complete growth cycle.

### 3.5. Difference of the Effects of Drought between Wheat Types

**Figure 5:**
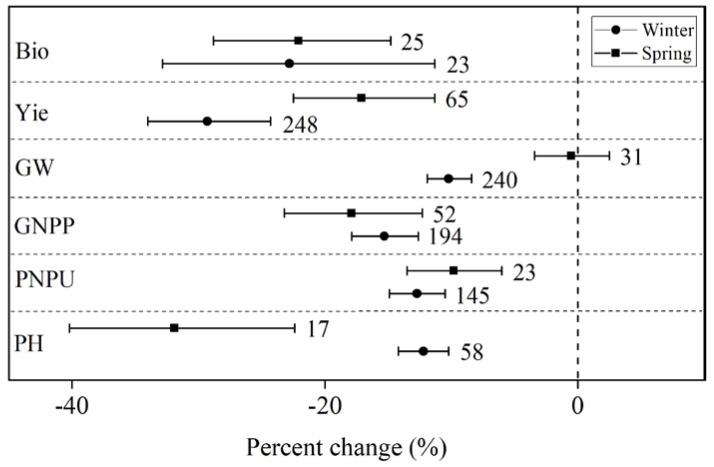
Effect of drought on winter and spring wheat. Numbers near the symbols specify the number of data points and the error bars indicate a 95% confidence interval. Abbreviations for the agronomic traits are described in Table 2.

Significant differences were found only in PH, GW, and yield between winter and spring wheat (see Figure 5). The spring wheat (−32.0%) had a much larger decrease in PH than that of the winter wheat (−12.0%). The GW of spring wheat showed a negligible decrease by 0.5%. However, the GW of winter wheat decreased by 10.0%. Little differences were shown in Bio, GNPP, and PNPU between winter and spring wheat. Spring wheat showed a larger decrease in GNPP than winter wheat did with regard to drought.

## 4. Discussion

### 4.1. How Crop Grain Yield is Reduced by Drought and How Yield Components Respond to Drought

The results suggested that there were differences among agronomic trait responses between rice and wheat under drought conditions (see Figure 1). Wheat demonstrated a 27.5% yield reduction and rice showed a 25.4% yield reduction despite the variations in the response observed in different studies (see Table 1). Variability of wheat and rice yields might be related to variations in plant physiological traits since different cereal species adopt different adaptation mechanisms to drought [12]. Yield loss has been attributed to the reduction in photosynthetic activity and lower supply of assimilates that support reproductive development and seed growth [8,25]. Some plants such as wheat can adapt to drought conditions via high osmotic adjustment and recovery after stress. Furthermore, wheat has the ability to shed their old leaves and maintain the carbon assimilation in their new, young leaves [28]. Rice is more sensitive to drought with a larger reduction in yield when compared to wheat (e.g., ~40% water deficit) [12]. Molecular genetics have discovered many quantitative trait loci that affect yield under drought conditions [29]. To avoid this large yield loss, the practical application of new molecular knowledge and tools for screening selection and improvement of rice germplasm for drought is required [30].

The most important component responsible for plant biomass reduction of wheat and rice was the decrease in grain yield, which was followed by a decrease in the PH. The yield reductions under drought conditions attributed to the decreases in yield components (e.g., PNPU, GW, weight of grains/spike, and biological yield) [21]. The component contributing the most to yield reduction varied between wheat and rice. For instance, PL and GNPP were the two most important components related to yield reduction in wheat (see Figure 1). However, GW and PNPU also showed significant decreases due to drought. This is consistent with previous studies where drought was shown to have a greater influence on grain number, which largely accounts for the decline in wheat yield [31]. Drought has an extremely adverse effect on meiosis and anthesis, which directly affects grain number. This causes a substantial reduction in grain yield [29]. Moreover, pollen becomes sterile when drought occurs during the early microspore stage of pollen development, which would reduce the grain number [32]. This phenomenon might indicate that the longer the panicle is, the more wheat grains it has. In contrast, the decrease in FP contributed the greatest addition to the rice yield reduction. It has been suggested that grain filling is damaged the most by drought over the complete rice growth cycle. Similar results have been reported in wheat influenced by elevated levels of O_3_ [25].

### 4.2. The Impact of Growth Stages, Stress Intensities, Rooting Environment, and Wheat Types on Crop Agronomic Traits in Response to Drought

**Growth stages.** Yield is basically the complex integration of the different growth stages. The negative influence of drought on the yield mainly depends on the sensitivity of the plant to drought during different growth stages [8]. For instance, drought occurring during the vegetative phase reduced the rice yield by 21–50.6% whereas severe drought during the flowering stage reduced the rice yield by 42–83.7% and moderate to severe drought during the whole reproductive stage reduced the rice yield by 51–90.6% (see Table 4). From the present meta-analysis study, rice was more sensitive to drought during the reproductive phases (e.g., blooming stage, filling stage, and maturity) than during the vegetative phases (e.g., tillering stage, jointing stage, and so on), which is shown in Figure 4. Previous studies have confirmed that greater yield reduction occurred during the reproductive stage [12] because crops barely recovered from the damage caused by drought. Drought during the vegetative phase limited carbohydrate synthesis for cell division and expansion via stomatal closure and partial inhibition of photosynthesis. Yet this was considered reparable [33]. The results obtained by Sarvestani et al. [34] proved the above theoretical conclusion that a water deficit during the flowering stage produced a lower rice yield by 50% when compared to a water deficit during the vegetative and grain filling stages. PNPU and GNPP were more adversely influenced during the vegetative phase (e.g., tillering stage and booting stage) than during the reproductive phase (see Figure 4) because the drought limited cell division for tillers, panicles, and grains. These results are supported by the results from Boonjung et al. [35] in which the reduction in rice yield of about 30% was due to reduced PNPU and the number of grains per panicle. In this case, the drought occurred during the vegetative stage.

However, some exceptions were found in wheat in response to drought. Wheat had similar sensitivity to drought during the vegetative and reproductive phases with no significant differences in agronomic traits found in the different stages (see Figure 4). This is in agreement with the global synthesis of wheat in response to drought [8,16]. Wheat is sensitive to drought during the vegetative phase (e.g., tillering stage) because drought limits the development of the root system, which results in decreased leaf area, leaf number per plant, leaf size, and leaf longevity [35]. Drought occurring during the tillering stage decreased wheat yield by 47.0%, which is much larger than during the booting stage and its 21.0% decrease [35]. This is supported by the results from a study that only considered normal tillers instead of a combination of late and normal tillers [13]. Wheat can produce late tillers when the early-season drought ends, which contribute to the final grain yield [13]. In the present meta-analysis, drought from the jointing stage to maturity reduced wheat yield significantly by decreasing GNPP, PL, and PNPU. When drought occurred in the mid-season (e.g., between the jointing stage and anthesis), its effect on wheat head size (i.e., the number of spikelet per spike) might be irreversible since late-emerging tillers would not contribute to yield [36]. It is more critical when drought occurred during the flowering and grain-filling stages than other stages and resulted in substantial yield reduction due to reduced rates of net photosynthesis owing to metabolic limitations (e.g., oxidative damage to chloroplasts and stomatal closure) and poor grain set and development [9,37]. The results show that post anthesis mild drought decreased wheat yields by 1–30% while a prolonged mild drought during the flowering and grain filling stages reduced grain yields by 58–92% [9].

**Drought stress intensity.** The severity of water stress variably affected yield-related traits in rice and wheat. The reductions of yield and its components in both rice and wheat under severe stress were significantly greater than that under mild stress (see Figure 2). In the present meta-analysis study, the current severe drought decreased wheat yield production by more than 40% and rice yield production by 30%. Considering the changing trends of climate change, drought is predicted to be more intense and frequent in many regions in the future [19] with global agricultural drought frequency and area increased by 50–200% in a relative sense during the 21st century so far [20]. Moderate and severe drought levels based on current climate standards will become the norm in the future [43] with a corresponding doubling of the spatial extent of severe soil moisture deficits and the frequency of short-term droughts at the end of the 21st century. Future droughts could produce a further lowering in yield and other agronomic traits when compared to the current drought. A previous study proved that moderate droughts (40% water reduction) and severe water deficits (>50% water reduction) caused different reductions in grain number, number of fertile ears per unit area, and number of grains per ear [44]. Therefore, severe droughts should be avoided during the tillering, booting, and flowering stages because drought limits the cell divisions for tillers, panicles, and grains [22,34]. These results might be explained by the oxidative damage to photo-assimilatory machinery under severe stress and the different reduced rates of carbon fixation [45]. A meta-analysis of plant leaf gas exchange showed that drought decreased net photosynthesis by 64% under severe stress, which was more than two times the decrease (−28.0%) under mild stress [46]. The damage from a severe drought to different growth stages is irreversible and unrecoverable.

**Rooting environment.** Root system architecture is one of the most important contributors to drought resistance in crops [8]. A well-developed root system is key to ensuring stable and high yields under drought [47] and the greater root length in deeper soil layers has been shown to increase yield by allowing more water extraction [48]. However, most of the root screening is restricted in artificial growth media, which limits inference to field grown crops [49]. The results from the present meta-analysis study showed that the rooting environment had a significant influence on wheat and rice yield. Rice grown in fields showed a larger decreased yield in response to drought when compared to rice grown in pots. Since rice is characterized by a shallower and more fibrous root system than wheat [50], it has limited water extraction below 60 cm [51]. The capacity of the pot (5~15 L) was acceptable for rice root development whereas rice grown in the field might have a competitive pressure of a water deficit from other plant individuals. The lateral root of rice showed greater development under drought [52], which would accelerate drought stress in 20 cm deep soil adverse to rice production. However, wheat grown in pots showed a larger decreased yield and yield-related traits than wheat grown in the field. This might be because the pot significantly limited the development of the wheat root systems, which were responsible for grain yield reductions in wheat because wheat roots could extend to an average of 113 cm deep and had less than 50% of their total root length in the top 20 cm of the soil [53]. Drought during the vegetative phase (e.g., tillering stage) limits root system development that leads to decreases in leaf area, leaf number per plant, leaf size, and leaf longevity [35].This aligns with the meta-analysis of wheat in response to elevated O_3_ that showed a larger decrease in biomass and yield for wheat grown in plots than those grown in the field [25].

**Wheat types.** It was assumed that the response of spring wheat to drought would be different to that of winter wheat. However, our meta-analysis assessment across all studies indicated that there was no significant difference between spring and winter wheat in terms of investigated parameters with an exception of PH and GW. Similar results were also found in a previous study [25] where there was no significant difference in yield and relative traits between spring and winter wheat in response to elevated O_3_ levels.

## 5. Conclusions

In the present meta-analysis study, we found that drought, which is an important limiting factor, decreased agronomic traits differently between wheat and rice among different growth stages. Rice demonstrated larger reductions in yield and in its components during the reproductive phase (e.g., blooming stage, filling stage, and maturity). Wheat exhibited similar reductions during the complete growth cycle. The rooting environment also affected the agronomic traits under drought conductions with opposite performance changes between wheat and rice. Based on these studies, future droughts could result in further decreases in yield and other agronomic traits than in the current drought. However, a drought is not the only element of global climate change that affects crop agronomic traits and it will interact with other factors such as precipitation, temperature, and increasing CO_2_ levels.

## Figures and Tables

**Table 1:** Yield losses in wheat and rice caused by drought.

Crop Species	Stress and Description	Yield Losses (%)	Reference
Wheat (*Triticum aestivum* L.)	Drought taken as 40–45% of soil natural water content (NWC: 100%)	57	Balla et al., 2011 [15]
	Drought taken as approximately 40% of soil water reduction	20.6	Daryanto et al., 2016 [13]
	Drought, water stress (∼40% water deficit)	<25	Daryanto et al., 2017 [12]
	Drought, tenth-percentile rainfall outcome	22	Tavk et al., 2014 [16]
	Drought and warming (1 °C warming)	33	Tavk et al., 2014 [16]
	Drought, the SPEI (Standardized Precipitation Evapotranspiration Index) denoting extreme dry (0.05 quantile)	4.4	Matiu et al., 2017 [9]
	Drought and high temperature, a 1 °C higher than average global temperature	9.2	Matiu et al., 2017 [9]
Rice (*Oryza sativa* L.)	Drying, soils dried beyond −20 kPa	22.6	Carrijo et al., 2017 [17]
	Drought, a period of water deficit when the soil water content declined below saturation	53–92	Lafitte et al., 2007 [18]
	Drought, water stress (∼40% water deficit)	>50	Daryanto et al., 2017 [12]

**Table 2:** List and description of response variables reported in the meta-analysis study.

Agronomic Traits		Growth Stage	
Variable	Description	Variable	Description
**Yie**	Yield, grain weight per plant or per m^2^	*Gc*	Drought during both the vegetative and reproductive phases or the complete growth cycle
**PH**	Plant height	*Fs*	Drought occurring at filling stage or grain-filling stage
**PL**	Panicle lengt or ear length	*Bls*	Drought occurring at blooming stage or from blooming stage to maturity
**GW**	1000-grain weight		
**FP**	Filled grains percentage or seed setting rate	*Hs*	Drought occurring at heading stage or from heading to maturity
**Bio**	Total biomass per plant at harvest		
**GNPP**	Grain number per panicle	*Bs*	Drought occurring at booting stage
**PNPU**	Panicle number per plant or m^2^	*Js*	Drought occurring at jointing stage or from jointing stage to maturity
		*Ts*	Drought occurring at tillering stage

**Table 3:** Heterogeneity (Q_b_) analysis of agronomic traits in rice and wheat.

Crop	Categorical Variable ^a^	Q_b_	N	*p*-Value	Crop	Categorical Variable ^a^	Q_b_	N	*p*-Value
Rice	Bio	226.76	57	0.0006	Wheat	Bio	55.01	55	0.4742
	Yie	1263.65	322	0.0002		Yie	482.68	317	0.0000
	GW	618.72	359	0.0050		GW	336.01	280	0.0240
	GNPP	853.73	288	0.0007		GNPP	573.16	251	0.0003
	PNPU	848.35	284	0.0078		PNPU	269.62	151	0.0008
	FP	1674.41	245	0.0006		PL	33.54	44	0.1226
	PL	1695.34	67	0.0001		PH	246.88	74	0.0092
	PH	470.23	151	0.0051		--	--	--	--

^a^ Abbreviations for the agronomic traits are described in Table 2, Q_b_: Heterogeneity, N: number of samples.

**Table 4:** Reductions in agronomic traits in rice caused by drought stress.

Growth Stage	Drought Stress	Yield Reduction (%) ^a^	Reference
Vegetative stage	Severe stress	50.6	Guan et al., 2010 [36]
Vegetative stage	Water stress	21	Sarvestani et al., 2008 [34]
Flowering stage	Severe stress	42	Pinheiro et al., 2000 [37]
Flowering stage	Severe stress	76.7–83.7	Puteh et al., 2013 [38]
Flowering stage	Water stress	50	Sarvestani et al., 2008 [34]
Flowering stage	Severe stress	>70	Shamsudin et al., 2016 [39]
Reproductive stage	Moderate stress	51–57	Dixit et al., 2014 [40]
Reproductive stage	Severe stress	70	Dixit et al., 2014 [40]
Reproductive stage	Moderate stress	90.6	Dixit et al., 2012 [41]
Reproductive stage	Severe stress	63.1	Dixit et al., 2012 [41]
Reproductive stage	Severe stress	74.5	Guan et al., 2010 [36]
Reproductive stage	Moderate to severe stress	51–60	Swamy et al., 2017 [42]

^a^ Yield reduction are percent change of yield loss effected by drought.

## Supplementary Materials

The following are available online at http://www.mdpi.com/1660-4601/15/5/839/s1, Table S1: The collected information used in the meta-analysis about effects of drought on rice and wheat.
