# Supplementary material for: Effect of Drought on Agronomic Traits of Rice and Wheat: A Meta-Analysis
Source: Int J Environ Res Public Health. 2018 Apr 24;15(5):839. doi: 10.3390/ijerph15050839 (PMC5981878; doi:10.3390/ijerph15050839)
Supplement: Supplementary file 1 [file ijerph-15-00839-s001.pdf]

International Journal of
Environmental Research and Public Health

MDPI

Supplementary Material

Effect of Drought on Agronomic Traits of Rice and Wheat: A Meta-Analysis

Jinmeng Zhang, Shiqiao Zhang, Min Cheng, Hong Jiang, Xiuying Zhang, Changhui Peng, Xuehe Lu, Minxia Zhang and Jiaxin Jin

Table S1. The collected information used in the meta-analysis about effects of drought on crop.

No.	Plant species	Variables	Reference
**1**	Rice	FP, PNPU, GNPP, GW, YIe	[1]
**2**	Rice	PH	[2]
**3**	Rice	PL, FP	[3]
**4**	Rice	PL, GNPP, GW	[4]
**5**	Rice	FP, PNPU, GW, YIe	[5]
**6**	Rice	PL, PH, FP, PNPU, GW, YIe	[6]
**7**	Rice	PNPU, BIo	[7]
**8**	Rice	PL, PH, PNPU, BIo	[8]
**9**	Rice	PH	[9]
**10**	Rice	PH, FP, GW	[10]
**11**	Rice	PH, FP, GNPP, GW	[11]
**12**	Rice	PH, FP, GW, YIe	[12]
**13**	Rice	PH	[13]
**14**	Rice	PH, FP, GW	[14]
**15**	Rice	PH, GW	[15]
**16**	Rice	PH, FP, GNPP, GW	[16]
**17**	Rice	FP, GW, YIe	[17]
**18**	Rice	FP, PNPU, GW, YIe	[18]
**19**	Rice	PL, FP, PNPU, GW, YIe	[19]
**20**	Rice	PNPU, GW, YIe	[20]
**21**	Rice	PH, PNPU, GW, YIe, BIo	[21]
**22**	Rice	PH, FP, PNPU, GNPP, GW, YIe	[22]
**23**	Rice	PL, PH, FP, PNPU, GW, YIe	[23]
**24**	Rice	PL, FP, PNPU, GW, YIe	[24]
**25**	Rice	FP, PNPU, GNPP, GW, YIe	[25]
**26**	Rice	PH, FP, PNPU, GW, YIe	[26]
**27**	Rice	FP, PNPU, GW, YIe	[27]
**28**	Rice	PH, FP, PNPU, GNPP, GW,YIe	[28]
**29**	Rice	FP, PNPU,GNPP, GW, YIe	[29]
**30**	Rice	FP, PNPU, GW, YIe	[30]
**31**	Rice	FP, PNPU,GW, YIe	[31]
**32**	Rice	PL, PNPU, GNPP, GW, YIe, BIo	[32]
**33**	Rice	GNPP, GW, YIe	[33]
**34**	Rice	FP, PNPU, GW, YIe	[34]
**35**	Rice	PNPU, YIe, BIo	[35]
**36**	Rice	PH, PNPU, GW, YIe	[36]
**37**	Rice	FP, PNPU, GNPP, GW, YIe, BIo	[37]

No.	Plant species	Variables	Reference
**38**	Rice	FP, PNPU, GNPP, GW, YIe, BIo	[38]
**39**	Rice	PNPU, GNPP, GW, YIe, BIo	[39]
**40**	Rice	FP, PNPU, GW, TIe	[40]
**41**	Rice	GW	[41]
**42**	Rice	PL, FP, PNPU, GNPP, GW, YIe	[42]
**43**	Rice	PL, FP,PNPU,GNPP, GW, YIe	[43]
**44**	Rice	PNPU, GNPP, GW, YIe	[44]
**45**	Rice	PNPU, GNPP, GW, YIe	[45]
**46**	Rice	PL, PH, FP,PNPU,GNPP, GW, YIe, BIo	[46]
**47**	Rice	FP, PNPU, NPP, GW, YIe	[47]
**48**	Rice	PL, PNPU, GNPP, GW, YIe	[48]
**49**	Rice	PL, FP, PNPU, GW, YIe	[49]
**50**	Rice	FP, PNPU, GNPP, GW, YIe	[50]
**51**	Rice	FP, PNPU, GNPP, GW, YIe	[51]
**52**	Rice	PL, FP, PNPU, GNPP, GW, YIe, BIo	[52]
**53**	Rice	FP, PNPU, GNPP, GW, YIe	[53]
**54**	Rice	PH, PNPU, YIe	[54]
**55**	Rice	PL, PH, FP,PNPU,GNPP, GW, YIe	[55]
**56**	Wheat	PH	[56]
**57**	Wheat	GNPP, GW	[57]
**58**	Wheat	PNPU, GNPP, GW, YIe	[58]
**59**	Wheat	PNPU, GNPP, GW, BIo	[59]
**60**	Wheat	GNPP, GW	[60]
**61**	Wheat	GNPP, GW	[61]
**62**	Wheat	PNPU, GNPP, GW, YIe	[62]
**63**	Wheat	GW, YIe, BIo	[63]
**64**	Wheat	GW	[64]
**65**	Wheat	YIe, BIo	[65]
**66**	Wheat	FP, YIe	[66]
**67**	Wheat	PH, PNPU, GW	[67]
**68**	Wheat	GNPP, YIe	[68]
**69**	Wheat	PH	[69]
**70**	Wheat	PH, PNPU, GW, YIe	[70]
**71**	Wheat	GW, YIe	[71]
**72**	Wheat	PNPU, GW, YIe	[72]
**73**	Wheat	PNPU, GW, YIe, BIo	[73]
**74**	Wheat	PNPU, GNPP, YIe	[74]
**75**	Wheat	PNPU, GNPP, GW, YIe	[75]
**76**	Wheat	GNPP, GW, YIe	[76]
**77**	Wheat	PNPU, GW, YIe, BIo	[77]
**78**	Wheat	YIe	[78]
**79**	Wheat	YIe	[79]
**80**	Wheat	PNPU, GNPP	[80]
**81**	Wheat	YIe	[81]
**82**	Wheat	PNPU, GNPP, GW	[82]
**83**	Wheat	PH, PNPU, GNPP, GW	[83]
**84**	Wheat	PL, PH	[84]
**85**	Wheat	GNPP, GW	[85]

No.	Plant species	Variables	Reference
**86**	Wheat	PL, PH, GNPP, GW, YIe	[86]
**87**	Wheat	PNPU, GNPP, GW, YIe	[87]
**88**	Wheat	PL, PH, GNPP, GW, YIe	[88]
**89**	Wheat	PNPU, GNPP, GW	[89]
**90**	Wheat	PNPU, GNPP, GW, YIe	[90]
**91**	Wheat	PH, GNPP, GW, YIe	[91]
**92**	Wheat	PL, PH, GNPP, GW, YIe	[92]
**93**	Wheat	YIe	[93]
**94**	Wheat	Bio	[94]
**95**	Wheat	GW, YIe	[95]
**96**	Wheat	PNPU, GNPP, GW, YIe, BIo	[96]
**97**	Wheat	PNPU, GW, YIe	[97]
**98**	Wheat	PL, GNPP, GW, YIe	[98]
**99**	Wheat	GNPP, GW, YIe	[99]
**100**	Wheat	PL, PH, PNPU, GNPP, GW, YIe	[100]
**101**	Wheat	YIe	[101]
**102**	Wheat	PNPU, GNPP, GW, YIe	[102]
**103**	Wheat	PL, PH, GNPP, GW, YIe	[103]
**104**	Wheat	PL, PH, PNPU, GNPP, GW, YIe	[104]
**105**	Wheat	GW	[105]
**106**	Wheat	PL, GNPP, GW, YIe	[106]
**107**	Wheat	PNPU, GNPP, GW, YIe, BIo	[107]
**108**	Wheat	PNPU, GNPP, GW, YIe	[108]
**109**	Wheat	PNPU, GNPP, GW	[109]
**110**	Wheat	PNPU, GNPP, GW, YIe	[110]
**111**	Wheat	PH, PNPU, GW	[111]
**112**	Wheat	YIe	[112]
**113**	Wheat	GW, YIe	[113]
**114**	Wheat	PNPU, GNPP, GW, YIe	[114]
**115**	Wheat	PNPU, GNPP, GW, YIe	[115]

YIe: Yield, grain weight per plant or per m^2; PH: Plant height; PL: Panicle length/Ear length; WG: 1000-grain weight; FP: Filled grains Percentage/seed setting rate; Bio: Total Biomass per plant at harvest; GNPP: Grain Number per Panicle; PNPU: Panicle Number per plant or m^2.

References

1. Yang, J.; Huang, D.; Hua, D.; Tan, G.; Zhang, J., Alternate wetting and moderate soil drying increases grain yield and reduces cadmium accumulation in rice grains. *Journal of the Science of Food & Agriculture* **2009,** 89, (10), 1728-1736.
2. Vikram, P.; Swamy, B. P. M.; Dixit, S.; Singh, R.; Singh, B. P.; Miro, B.; Kohli, A.; Henry, A.; Singh, N. K.; Kumar, A., Drought susceptibility of modern rice varieties: an effect of linkage of drought tolerance with undesirable traits. *Scientific Reports* **2015,** 5, (14799).
3. Rang, Z. W.; Jagadish, S. V. K.; Zhou, Q. M.; Craufurd, P. Q.; Heuer, S., Effect of high temperature and water stress on pollen germination and spikelet fertility in rice. *Environmental & Experimental Botany* **2011,** 70, (1), 58-65.
4. Wu, N.; Guan, Y.; Shi, Y., Effect of Water Stress on Physiological Traits and Yield In Rice Backcross Lines after Anthesis. *Energy Procedia* **2011,** 5, (5), 255-260.
5. Yu, M.; Wang, X.; Duan, Y.; Tian, X.; Jia, Y.; Zhao, H., Effect of Drought Stress at Tillering Stage on Photosynthetic Characteristics and Yield Formation of Cold-region Rice. *Journal of Nuclear Agricultural Sciences* **2017,** 31, (9), 1794-1802.

6. Wang, S. M.; Huang, Y. L.; Zhu, Q. S.; Wang, W. L.; Zhang, D. W.; Yang, Q. J., Effects of water stress on economic characters of water-saving rice variety Luhan No. 1. *Agricultural Science & Technology* **2011,** 12, (10), 1453-1456,1460.
7. Kato, Y.; Kamoshita, A.; Yamagishi, J., Evaluating the resistance of six rice cultivars to drought: restriction of deep rooting and the use of raised beds. *Plant & Soil* **2007,** 300, (1-2), 149-161.
8. Ghneimherrera, T.; Selvaraj, M. G.; Meynard, D.; Fabre, D.; Peña, A.; Romdhane, W. B.; Saad, R. B.; Ogawa, S.; Rebolledo, M. C.; Ishitani, M., Expression of the Aeluropus littoralis AlSAP Gene Enhances Rice Yield under Field Drought at the Reproductive Stage. *Frontiers in Plant Science* **2017,** 8, (994), 1-14.
9. Ma, X.; Feng, F.; Wei, H.; Mei, H.; Xu, K.; Chen, S.; Li, T.; Liang, X.; Liu, H.; Luo, L., Genome-Wide Association Study for Plant Height and Grain Yield in Rice under Contrasting Moisture Regimes. *Frontiers in Plant Science* **2016,** 7.
10. Nurul Amalina, M. Z.; Ismail, M. R.; Puteh, A.; Mahmood, M.; Islam, M. R., Impact of cyclic water stress on growth, physiological responses and yield of rice (Oryza sativa L.) grown in tropical environment. *Ciência Rural* **2014,** 44, (2), 155-156.
11. Shao, G.; Cui, J.; Yu, S. E.; Lu, B.; Brian, B. J.; Ding, J.; She, D., Impacts of controlled irrigation and drainage on the yield and physiological attributes of rice. *Agricultural Water Management* **2015,** 149, 156-165.
12. Kijoji, A. A.; Nchimbi-Msolla, S.; Kanyeka, Z. L.; Serraj, R.; Henry, A., Linking root traits and grain yield for rainfed rice in sub-Saharan Africa: Response of Oryza sativa × Oryza glaberrima introgression lines under drought. *Field Crops Research* **2014,** 165, 25-35.
13. Venuprasad, R.; Impa, S. M.; Gowda, R. P. V.; Atlin, G. N.; Serraj, R., Rice near-isogenic-lines (NILs) contrasting for grain yield under lowland drought stress. *Field Crops Research* **2011,** 123, (1), 38-46.
14. Guan, Y. S.; Serraj, R.; Liu, S. H.; Xu, J. L.; Ali, J.; Wang, W. S.; Venus, E.; Zhu, L. H.; Li, Z. K., Simultaneously improving yield under drought stress and non-stress conditions: a case study of rice (Oryza sativa L.). *Journal of Experimental Botany* **2010,** 61, (15), 4145-4156.
15. Sarvestani, Z. T.; Pirdashti, H.; Sanavy, S. A.; Balouchi, H., Study of water stress effects in different growth stages on yield and yield components of different rice (Oryza sativa L.) cultivars. *Pakistan Journal of Biological Sciences Pjbs* **2008,** 11, (10), 1303-1309.
16. Pantuwan, G.; Fukai, S.; Cooper, M.; Rajatasereekul, S.; O'Toole, J. C., Yield response of rice (Oryza sativa L.) genotypes to different types of drought under rainfed lowlands - Part 1. Grain yield and yield components *Field Crops Research* **2002,** 73, (01), 181-200.
17. Defu, L.; Jincai, L.; Fengzhen, W., Effects of Water-stress on Some Physiologic Characteristics and Economic Yield at Jointing and Pregnancy Stage of Dry Cultivation Rice. *Journal of Anhui Agricultural Sciences* **2005,** 33, (7), 1166-1167,1169.
18. Jiang, X. H.; Li, G. H.; Wang, S. H.; Luo, D. Q.; Zhou, W. J.; Liu, Z. H.; Li, M.; Ding, Y. F., Effect of drought stress at different growth stages on grain yield of indica hybrid rice. *Journal of Nanjing Agricultural University* **2015,** 38, (2), 173-181.
19. Wang, Z. J.; Chen, Y. J.; Xie, C. H.; Yang, G. T., Effect of water stress on photosynthesis and yield character of hybrid rice at different stages. *Agricultural Research in the Arid Areas* **2008,** 26, (6), 138-141.
20. Wang, C.; Wang, B.; Zhang, W.; Zhao, L.; Zhao, X.; Gao, L.; Hou, W., Effects of drought stress at different growth stages on grain yield and milling quality of rice. *Chinese Journal of Rice Science* **2007,** 21, (6), 643-649.
21. Shao, X.; Liu, H.; Du, Z.; Yang, J.; Meng, F.; Ma, J., Effects of Water Disposal on Growth and Yield of Rice. *Journal of Soil & Water Conservation* **2007,** 21, (1), 193-196.
22. Zhang, Y.; Zhu, D.; Lin, X.; Chen, H., Effects of water stress on rice growth and yield at different growth stages. *Agricultural Research in the Arid Areas* **2005,** 23, (2), 48-53.
23. Xie, W.; Zhang, W.; Shi, H.; He, M.; Zhang, L.; Wang, Z., Study on the influence of soil water stress on rice yield and taste quality in different periods. *Liaoning Agricultural Sciences* **2007**, (2), 30-33.

24. Ke, C., Effect of different water treatment on rice growth, yield and quality. *Huazhong Agricultural University* **2010**.
25. Wang, W. X.; Liu, X. J.; Tian, Y. C.; Yao, X.; Gao, W. X.; Zhu, Y., Effects of different soil water treatments on photosynthetic characteristics and grain yield in rice. *ACTA ECOLOGICA SINICA* **2012,** 32, (22), 7053-7060.
26. Lu, J., Effects of sustained soil water stress on growth, development and yield components of two rice cultivars. *Journal of Jiangsu Agricultural College* **1998,** 19, (2), 43-48.
27. Yang, X.; Wang, B.; Chen, L.; Cao, C.; Li, P., Effects of Drought Stress on Rice Physiological Traits and Grain Yield at Heading Stage. *China Rice* **2015,** 04, (21), 138-141.
28. Cong, X. H.; Shi, F. Z.; Xin-Min, R.; Luo, Z. X., Effect of high temperature and drought on yield and grain quality of different indica rice varieties during heading periods. *Journal of Henan Agricultural University* **2014,** 48, (6), 667-673.
29. Chi, D.; Tong, Y.; Chen, T.; Zheng, J.; Chen, W.; Tai, E., Effects of Water Stress Coupling in Different Growth Stage on Rice Yield and Water Productivity. *Journal of Shenyang Agricultural University* **2016,** 01, (47), 71-77.
30. Zhou, G. S.; Cai-Guo, X. U.; Jin, D. M.; Cui, K. H.; Cao, C. G.; Cai, M. L.; Luo, B. S., Effects of Water Saving Treatment at Tillering Stage on Biological Characteristics of Rice. *Scientia Agricultura Sinica* **2005,** 38, (09), 1767-1773.
31. Yao, L.; Zheng, H. B.; Liu, J. X.; Hui, H. E.; Huang, H., Effects of Water Stress at Tillering Stage on Rice Growth and Development and Yield of Rice under Different Cultivation Modes. *Crop Research* **2014,** 28, (5), 455-460.
32. Shao, X.; Changchun, R., Effects of water stress on growth and yield or rice in tillering stage. *Journal of Jilin Agricultural University* **2005,** 26, (3), 237-241.
33. Zhu, B.; Yu, W.; Xiao, X.; Wu, J.; Chen, J.; Zhou, W.; Shu, Y., Study on the effect of drought on the growth and yield of rice. *Journal of Guizhou Agricultural Sciences* **1990**, (5), 37-41.
34. Zhang, W. X.; Zhu, D. F.; Lin, X. Q.; Zhang, Y. P.; Chen, H. Z.; Zhu, Z. W., The effect of drought stress on yield and panicle traits of different type super rice varieties during panicle initiation to grain filling stage. *Agricultural Research in the Arid Areas* **2010,** 28, (1), 255-260.
35. Wang, C.; Zhao, L.; Wang, B.; Zhang, W.; Zhao, X.; Gao, L.; Hou, W.; Yu, Y., Effect of Water Stress of Soil on Growing Characteristics and Physiological Index of Rice(Oryza sativa). *Journal of Agriculture* **2014,** 4, (1), 4-14.
36. Duan, S. M.; Yang, A. Z.; Huang, Y. D.; Wen-Ge, W. U.; You-Zun, X. U.; Chen, G.; University, A. A., Effects of Drought Stress on Growth and Physiological Feature and Yield of Various Rice Varieties. *Journal of Nuclear Agricultural Sciences* **2014,** 28, (6), 1124-1132.
37. Chen, L., The effect of drought stress on rice leaf's photosynthesis, rice grain yield and rice quality. *Huanzhong Agricultural University* **2015**.
38. Zhao, B.; Ye, Y.; Chen, X.; Wang, Z.; Yang, J.; Zhu, Q.; Zhang, H., Effect of Water Stress During Grain Fil ling on the Grain Yield and Qual ity of Two-l ine Hybrid Rice. *Journal of Yangzhou University* **2004,** 25, (01), 46-50.
39. Zhang, R. Z.; Shao, X. W.; Tong, S. Y.; Zhen-Yu, D. U.; Yang, M., Effects of Water Stress on Yield Composition and Yield of Rice in Blooming Stage. *Journal of Jilin Agricultural University* **2006,** 28, (1), 1-7.
40. Ding, Y.; Huang, W.; Wang, J.; Chang, H.; Wang, J., Effect of dry cultivation on the yield and yield component factors of rice. *Agricultural Reseach in the Arid Areas* **2002,** 20, (4), 50-54.
41. Zhou, G.; Jin, D.; Mei, F., Effects of Drought on Rice Grain Indices at Booting Stage. *Journal of Huazhong Agricultural* **2003,** 22, (3), 219-222.
42. Wang, Z. J.; Chen, Y. J.; Xie, C. H.; Tao, S. S.; Yang, G. T., Research on Physiology and Yield Character of Hybrid Rice B You 827 Cultivated in Different Water Condition. *Journal of Southwest University of Science & Technology* **2008,** 23, (4), 78-81.
43. Hua, F. U.; Liu, J.; Huang, Q.; Xiu-Ming, L. U.; Liu, H. Z., Effect of water stress on no-tillage cast-transplanted rice. *Journal of Zhangkai Aguotechaical College* **2000,** 13, (2), 18-22.

44. Wu, L., Effect of water stress on yield structure and yield of rice. *Hebei Water Technology* **1996,** 17, (3), 8-12.
45. Suo, W. W.; Fu, L.-D.; Wang, B. L.; Xu, J.-S.; Wang, Y.; Sui, X., Effect of Water Stress on Evapotranspiration and Yield of Rice. *North Rice* **2008,** 38, (5), 17-22.
46. Wang, C.; Wang, B.; Zhang, W.; Zhao, L.; Zhao, X.; Gao, L., Effects of water stress of soil on rice yield and quality. *Acta Agronomica Sinica* **2006,** 32, (1), 131-137.
47. Li, H.; Zhang, Y.; Yang, Y.; Zhu, D., Effects of soil moisture on superficial roots and yield of rice. *Agricultural Research in the Arid Areas* **2006,** 24, (01), 96-99.
48. Gao, Y.; Yang, T. T.; Bao, G. W.; Duan, S. M.; Yang, A. Z., Effects of Drought Stress at Booting Stage on Properties and Yield of Hybrid Rice. *Journal of Anhui Science & Technology University* **2015,** 29, (1), 19-22.
49. Wu, L. M.; Chen, D. W.; Lu, L. W.; Huang, M.; Zhang, Y.; Xu, S. H.; Tang, G. R.; Tian, L.; Jiang, L. G., Effects of water stress and re-watering at booting and grain filling stages on physiology and yield of Guiliangyou 2. *Journal of Southern Agriculture* **2014,** 45, (6), 955-960.
50. Xiao, X.; Deng, Y.; Li, Y.; Duan, L.; Zhou, Y.; Wang, J.; Fu, G., Effects of Water Stress on Physiological Characteristics and Yield of Rice. *Journal of Anhui Agricultural Sciences* **2009,** 37, (8), 3395-3398.
51. Feng, X. L.; Zhong, K. Y.; Tang, X. R.; Yuan, L. I.; Liu, Q., Effect of Water Stress in Rice Booting Stage on Sword Leaf Stomatal Traits,CAT Activity and Yield. *Journal of Irrigation & Drainage* **2014,** 33, (1), 135-137.
52. Shao, X. W.; Zhang, R. Z.; Chun-Yan, Q. I.; Tong, S. Y.; Yang, M., Effects of Water Stress on Growth and Yield of Rice in Jointing-Booting Stage. *Journal of Jilin Agricultural University* **2004,** 26, (3), 237-241.
53. Guo, H.; Ma, J.; Li, S. X.; Li, M.; Zhu, P.; Chen, Y., Effects of water stress on partial physiological characteristics and yield compensation in rice at booting stage. *Journal of Southern Agriculture* **2013,** 44, (9), 1448-1454.
54. Chen, W.; Yao, X.; Cai, K.; Chen, J., Silicon alleviates drought stress of rice plants by improving plant water status, photosynthesis and mineral nutrient absorption. *Biological Trace Element Research* **2011,** 142, (1), 67-76.
55. Wang, H.; Xu, G.; Ma, J.; Li, X.; Zhang, R., Effects of water stress on growth,development and yield of rice. *CHINA SEED INDUSTRY* **2009**, (1), 47-49.
56. Li, P.; Chen, J.; Wu, P., Agronomic Characteristics and Grain Yield of 30 Spring Wheat Genotypes under Drought Stress and Nonstress Conditions. *Agronomy Journal* **2011,** 103, (6), 1619-1628.
57. Guóth, A.; Tari, I.; Gallé, Á.; Csiszár, J.; Pécsváradi, A.; Cseuz, L.; Erdei, L., Comparison of the Drought Stress Responses of Tolerant and Sensitive Wheat Cultivars During Grain Filling: Changes in Flag Leaf Photosynthetic Activity, ABA Levels, and Grain Yield. *Journal of Plant Growth Regulation* **2009,** 28, (2), 167-176.
58. Li, X.; Topbjerg, H. B.; Jiang, D.; Liu, F., Drought priming at vegetative stage improves the antioxidant capacity and photosynthesis performance of wheat exposed to a short-term low temperature stress at jointing stage. *Plant & Soil* **2015,** 393, (1-2), 307-318.
59. Qin, N.; Xu, W.; Hu, L.; Li, Y.; Wang, H.; Qi, X.; Fang, Y.; Hua, X., Drought tolerance and proteomics studies of transgenic wheat containing the maize C4 phosphoenolpyruvate carboxylase (PEPC) gene. *Protoplasma* **2015,** 253, (6), 1-10.
60. Bijanzadeh, E.; Emam, Y., Effect of defoliation and drought stress on yield components and chlorophyll content of wheat. *Pakistan Journal of Biological Sciences Pjbs* **2010,** 13, (14), 699-705.
61. Dolatpanah, T.; Roustaii, M.; Ahakpaz, F.; Mohebalipour, N., Effect of Drought Stress on Grain Yield and Yield Components of Winter and Facultative Barley Genotypes in Maragheh Region. *Seed & Plant Improvment Journal* **2013**.
62. Ma, S.; Duan, A.; Ma, S.; Yang, S., Effect of Early-Stage Regulated Deficit Irrigation on Stem Lodging Resistance, Leaf Photosynthesis, Root Respiration and Yield Stability of Winter Wheat under Post-Anthesis Water Stress Conditions. *Irrigation and Drainage* **2016,** 65, (5), 673-681.

63. Nagarajan, S.; Rane, J.; Maheswari, M.; Gambhir, P. N., Effect of post-anthesis water stress on accumulation of dry matter, carbon and nitrogen and their partitioning in wheat varieties differing in drought tolerance. *J[Agronomy + Crop Science* **1999,** 183, 129-136.
64. Li, A.; Hou, Y.; Trent, A., Effects of elevated atmospheric CO_2 and drought stress on individual grain filling rates and durations of the main stem in spring wheat. *Agricultural & Forest Meteorology* **2001,** 106, (4), 289-301.
65. Ewert, F.; Rodriguez, D.; Jamieson, P.; Semenov, M. A.; Mitchell, R. A. C.; Goudriaan, J.; Porter, J. R.; Kimball, B. A.; Pinter, P. J.; Manderscheid, R.; Weigel, H. J.; Fangmeier, A.; Fereres, E.; Villalobos, F., Effects of elevated CO2 and drought on wheat: testing crop simulation models for different experimental and climatic conditions. *Agriculture, Ecosystems & Environment* **2002,** 93, (1), 249-266.
66. Liu, E. K.; Mei, X. R.; Yan, C. R.; Gong, D. Z.; Zhang, Y. Q., Effects of water stress on photosynthetic characteristics, dry matter translocation and WUE in two winter wheat genotypes. *Agricultural Water Management* **2016,** 167, 75-85.
67. Gizaw, S. A.; Garland-Campbell, K.; Carter, A. H., Evaluation of agronomic traits and spectral reflectance in Pacific Northwest winter wheat under rain-fed and irrigated conditions. *Field Crops Research* **2016,** 196, 168-179.
68. Abdullah, A. S.; Aziz, M. M.; Siddique, K. H. M.; Flower, K. C., Film antitranspirants increase yield in drought stressed wheat plants by maintaining high grain number. *Agricultural Water Management* **2015,** 159, 11-18.
69. Hubbard, M.; Germida, J. J.; Vujanovic, V., Fungal endophytes enhance wheat heat and drought tolerance in terms of grain yield and second-generation seed viability. *Journal of Applied Microbiology* **2014,** 116, (1), 109.
70. Khakwani, A. A.; Dennett, M. D.; Munir, M.; Abid, M., Growth and yield response of wheat varieties to water stress at booting and anthesis stages of development. *Pakistan Journal of Botany* **2012,** 44, (3), 879-886.
71. Li, Y. F.; Wu, Y.; Hernandez-Espinosa, N.; Peña, R. J., Heat and drought stress on durum wheat: Responses of genotypes, yield, and quality parameters. *Journal of Cereal Science* **2013,** 57, (3), 398-404.
72. Weldearegay, D. F.; Yan, F.; Jiang, D.; Liu, F., Independent and Combined Effects of Soil Warming and Drought Stress During Anthesis on Seed Set and Grain Yield in Two Spring Wheat Varieties. *Journal of Agronomy & Crop Science* **2012,** 198, (4), 245–253.
73. Chen, R.; Cheng, W.; Cui, J.; Liao, J.; Fan, H.; Zheng, Z.; Ma, F., Lateral spacing in drip-irrigated wheat: The effects on soil moisture, yield, and water use efficiency. *Field Crops Research* **2015,** 179, 52-62.
74. Zhang, X.; Jian, C.; Bernd, W.; Liu, F.; Dai, T.; Cao, W.; Dong, J., Multiple Heat and Drought Events Affect Grain Yield and Accumulations of High Molecular Weight Glutenin Subunits and Glutenin Macropolymer in Wheat. *Journal of Cereal Science* **2013,** 57, (1), 134-140.
75. Arguello, M. N.; Mason, R. E.; Roberts, T. L.; Subramanian, N.; Acuña, A.; Addison, C. K.; Lozada, D. N.; Miller, R. G.; Gbur, E., Performance of soft red winter wheat subjected to field soil waterlogging: Grain yield and yield components. *Field Crops Research* **2016,** 194, 57-64.
76. Xu, H.; Biswas, D. K.; Li, W. D.; Chen, S. B.; Zhang, L.; Jiang, G. M.; Li, Y. G., Photosynthesis and yield responses of ozone-polluted winter wheat to drought. *Photosynthetica* **2007,** 45, (4), 582-588.
77. Guan, Y.; Qiao, Z.; Du, J.; Du, Y., Root carbon consumption and grain yield of spring wheat in response to phosphorus supply under two water regimes. *Journal of Integrative Agriculture* **2016,** 15, (7), 1595-1601.
78. Ehdaie, B.; Layne, A. P.; Waines, J. G., Root system plasticity to drought influences grain yield in bread wheat. *Euphytica* **2012,** 186, (1), 219-232.
79. El-Hendawy, S. E.; Hassan, W. M.; Al-Suhaibani, N. A.; Schmidhalter, U., Spectral assessment of drought tolerance indices and grain yield in advanced spring wheat lines grown under full and limited water irrigation. *Agricultural Water Management* **2017,** 182, 1-12.

80. Foulkes, M. J.; Scott, R. K.; Sylvesterbradley, R., The ability of wheat cultivars to withstand drought in UK conditions: resource capture. *Journal of Agricultural Science* **2001,** 137, (1), 1-16.
81. Mahmood, N.; Ahmad, B.; Hassan, S.; Bakhsh, K., Wheat yield response to physiological limitations under water stress condition. *Journal of Animal and Plant Sciences* **2012,** 22, (3), 773-780.
82. Liu, Z. G.; Sun, J. S.; Zhang, J. Y.; Wang, J. L.; Xiao-Dong, L. I.; Liu, X. F., Effect of Drought at Different Growing Stages on Yield and Quality Characteristics of Strong-gluten Wheat. *Journal of Triticeae Crops* **2008,** 28, (5), 877-882.
83. Li, X.; Ni, S., Agronomic and Physiological Characterization of the Wide Adaptable Wheat Cultivar Zhongmai 175 Under Two Different Irrigation Conditions. *Scientia Agricultura Sinica* **2015,** 48, (21), 4374-4380.
84. Guan, Z.; Huo, Y., Effect of Drought on Growth and Development of Wheat. *Anhui Agri.Sci.Bull* **2006,** 12, (2), 48-49.
85. Zhang, J.; Liu, H.; Xiao-Ping, L. I.; Min, L. U.; Sun, S. G.; Jia-Ni, S. U.; Liu, S. H.; Chen, X. H.; Aamp, N.; University, F., Effect of drought on leaf physiological parameters and yield of wheat at booting stage. *Agricultural Research in the Arid Areas* **2014,** 32, (3), 1-8.
86. Zhang, X.; Feng, W.; Wu, S.; Tian, W.; Zhang, Y.; Yang, H.; Li, T., Effect of Drought Stress on WUE and Yield Traits of Different Winter Wheat Cultivars. *Journal of Henan Agricultural Sciences* **2012,** 41, (8), 21-25.
87. Zhang, Y. Q.; Lin, Q.; Liu, J. B.; Zhang, H. S.; Zhao, C. X., Effects of Drought Stress on Photosynthetic Characteristics and Yield of Different Fertilizer and Water Types of Wheat. *Journal of Triticeae Crops* **2011,** 31, (4), 724-730.
88. Haixia, X.; Wei, L.; Xiyong, C.; Zhongdong, D.; Yang, L.; Dangqun, C., Drought Stress Effect on Agronomic Traits of Wheat. *Chinese Agricultural Science Bulletin* **2008,** 24, (3), 125-129.
89. Yuan, R.; Ping, L. I.; Xiaoxue, H. U.; Zong, Y.; Sun, M.; Dong, Q.; Hao, X., Effects of Drought Stress on Physiological Characteristics and Yield of Wheat. *Journal of Shanxi Agricultural Sciences* **2016,** 44, (10), 1446-1449,1466.
90. Wu, J.; Wang, Z.; Li, Y.; Zhang, Y., Characteristics of yield formation and water use in different drought tolerance cultivars of winter wheat under drought stress. *Journal of China Agricultural University* **2015,** 20, (6), 23-35.
91. Guan, J.; Ma, C.; Li, G., The change of biomass of the root and shoot under drought stress and its relation with drought-resistance in wheat. *Journal of Agricultural University of Hebei* **2004,** 27, (1), 1-5.
92. Li, Y. C.; Zhang, C. Y.; Pang, Q. H.; Ren, M. Q., Study on wheat resistance to drought in the different growing stages under drought stress. *Southwest China Journal of Agricultural Sciences* **2008,** 21, (3), 621-624.
93. Yuan, Y.; Deng, X., Effect of deficit and rewatering on wheat photosynthesis and yield. *Acta Botanica Boreali-occidentalia Sinica* **2004,** 24, (7), 1250-1254.
94. Li, Y., Effects of Continuous Drought on Grain Filling Characteristics of Wheat with Different Yield Potential. *Acta Agriculturae Boreali-occidentalis Sinica* **1997,** 6, (4), 47-50.
95. Shi, G.; Guo, X.; Liu, S.; Chen, M.; Guozhan, F. U.; Luoyang, Effect of Water Stress on Flag Leaves Senescence and Grain Yield of Winter Wheat in Grain Filling Stage. *Acta Agriculturae Boreali-occidentalis Sinica* **1999,** 8, (2), 26-29.
96. Zhang, C.; Deng, X. P.; Chen, W., Effect of sucrose accumulation and yield components on winter wheat flag leaf under drought or re-water during filing stage. *Journal of Northwest A & F University* **2011,** 39, (6), 68-74, 81.
97. Pengli, Y.; Chenyeng, W.; Hongfang, L.; Weixing, L.; Geng, M.; Qiang, W.; Yangyang, H., Effect of Heat and Drought Stress on Starch Accumulation During Grain Filling Stage. *Journal of Triticeae Crops* **2016,** 36, (11), 1489-1496.
98. WU, J.; Jin, Y.; Xie, Y., Effect of Drought on Physiology, Yield and Quality of Wheat in Later Stage. *Journal of Nenan Agricultural College* **1983,** 2, (2), 40-44.
99. Fan, X. M.; Jiang, D.; Dai, T. B.; Jing, Q.; Cao, W. X., Effects of post-anthesis drought and waterlogging on the quality of grain formation in different wheat varieties. **2004,** 28, (5), 680-685.

100. Pan, Z.; Wang, Y., Effect of Water Saving Irrigation on Character and Yield of Winter Wheat in Watershed Region of Jianghuai. *Modern agricultural science and technology* **2011,** 23, 76-77.
101. Liu, L. P.; Zhu, O.; Lan-Fang, W. U.; Fa-Dong, L. I.; Liu, P. F., Effects of phased drought and re-watering on the photosynthetic characteristics and grain yield of winter wheat. *Chinese Journal of Ecology* **2012,** 31, (11), 2797-2803.
102. Yan, P.; Mei, X.; Cao, L.; Yang, S.; Zhang, R., Effect of Water-saving Cultivation on Yield Components and Quality of Wheat. *Journal of Jiangsu Agricultural Science* **2004,** 6, 32-34.
103. Di, S., Analyze the Influence of Drought Stress on Agronomic Traits of Wheat. *China New Technologies and Products* **2014,** 4, 176-176.
104. Song, N.; Huang, X. Q.; Sun, J. S.; Liu, Z. G.; Wang, J. L., Effects of water stress on yield and quality of potted winter wheat. *Journal of Irrigation & Drainage* **2008,** 27, (3), 476-479.
105. Lv, D.; Yang, J.; Li, L.; Guo, X.; Sun, X., Correlative Study on the Physiological Reaction and Yield Performance of Wheat Varieties Stressed by Water Deficiency. *Acta Agriculturae Universitatis Henanensis* **1994,** 28, (3), 230-235.
106. Limei, H.; Yongjiu, Z.; Shuqi, W.; Fei, Y., Study on Infuence of Water -force and Fertilizing to Economic characters and Yield of Wheat. *Jilin Agriculturalences* **1998**, (2), 19-22.
107. Zhang, W.; Qian, X.; Yinyin, L. I.; Yunji, X. U.; Wang, Z.; Yang, J., Effect of Soil Drought on the Physiological Traits and Grain Yield of Wheat. *Journal of Triticeae Crops* **2016,** 36, (4), 491-500.
108. Song, X. Y.; Shuang, W. U.; Zhang, H. S.; Lin, Q.; Ping, M. U., Effect of Soil Water Stress on Physiological Characteristics in Different Winter Wheat Cultivars. *Acta Agriculturae Boreali-Sinica* **2014,** 29, (2), 174-180.
109. Dequan, L.; Yiqin, Z.; Qi, Z.; Bingsong, C., Effect of soil water stress in water status, phoyosynthsis and yield of wheat with drought resistance. *Journal of Shandong Agricultural University* **1992,** 23, (2), 125-130.
110. Liu, G.; Zhang, R.; Lu, J.; Gu, J., Relationship Between Yield and indices Determining Drought-Resistance in Winter Wheat. *Journal of Agricultural University of Hebei* **1995,** 18, (1), 10-14.
111. Zhang, X.; Chen, X.; Sang, T.; Xiao, L.; WANG, L.; Gao, Y.; Cao, P., Effect of the Drought in the Middle-later Period of Wheat Growth on its Growth and Yield. *Journal of Anhui Agricultural Sciences* **2000,** 28, (4), 452-453.
112. Hu, J.; Cao, W.; Jiang, D.; Luo, W., Quantification of water stress factor for crop growth simulation I. Effects of drought and waterlogging stress on photosynthesis, transpiration and dry matter partitioning in winter wheat. *Acta Agronomica Sinica* **2004,** 30, (4), 315-320.
113. Shi, H.; Li, C., Effctts of Irrigation at Middle Final Stage on the Grain Yield and Nutrient Quality of Winter Wheat. *Journal of Henan Vocation-technical Teachers College* **1989,** 17, (3-4), 108-112.
114. Le, Z.; Liao, R.; Liu, J.; Lu, J.; Bai, Y.; Liang, H.; An, S.; Huang, H., Effects of water stress on stem,plant and yield of winter wheat over North China plain. *Journal of Meteorology & Environment* **2014,** 30, (6), 120-124.
115. Wang, H.; Yu, H.; Deng, G.; Zheng, D.; Liu, Z., Studies on Effects of Wheat Water Stress at Different Development Stages on Yield and Drought Determination. *Acta Agriculturae Boreali—sinica* **1989,** 4, (4), 1-5.
